# A self‐applied valid scale for rapid tracking of household food insecurity among pregnant women in Sri Lanka

**DOI:** 10.1111/mcn.13165

**Published:** 2021-03-17

**Authors:** Thilini C. Agampodi, Amber Hromi‐Fiedler, Suneth B. Agampodi, Gayani S. Amarasinghe, Nuwan D. Wickramasinghe, Imasha U. Jayasinghe, Ayesh U. Hettiarachchi, Rafael Perez‐Escamilla

**Affiliations:** ^1^ Department of Community Medicine, Faculty of Medicine and Allied Sciences Rajarata University of Sri Lanka Anuradhapura Sri Lanka; ^2^ Department of Social and Behavioral Sciences Yale School of Public Health New Haven Connecticut USA

**Keywords:** COVID‐19, household food insecurity, measurement scale, pregnancy, rapid surveys, Sri Lanka, validity

## Abstract

Rapid household food insecurity (HFI) tracking has been identified as a priority in the context of the COVID‐19 pandemic and its aftermath. We report the validation of the Latin American and Caribbean Food Security Scale (*Escala Latinoamericana y Caribena de Seguridad Alimentaria* [ELCSA]) among pregnant women in Sri Lanka. The eight‐item adult version of the ELCSA was translated from English to Sinhala and Tamil. Cognitive testing (on 10 pregnant women and five local experts) and psychometric validation of the self‐administered HFI tool were conducted among pregnant women (*n* = 269) attending the special clinics of the Rajarata Pregnancy Cohort (RaPCo) in Anuradhapura in February 2020. We assessed the psychometric properties and fit using a one parameter logistic model (Rasch model analysis) using STATA Version 14 and WINSTEP software Version 4.3.4. Concurrent validity was tested using psychological distress. The scale was internally consistent (Cronbach's alpha = 0.79) and had a good model fit (Rasch items infit statistic range: 0.85 to 1.07). Item 8 (‘did not eat for the whole day’) was removed from the model fit analysis, as it was not affirmed by respondent. Item severity scores ranged from −2.15 for ‘not eating a diverse diet’ to 4.43 for ‘not eating during the whole day’. Concurrent validity between HFI and psychological distress was confirmed (*r* = 0.15, *p* < 0.05). The self‐applied version of ELCSA‐pregnancy in Sri Lanka (ELCSA‐P‐SL) is a valid and feasible valid tool. We recommend it to track HFI among pregnant women in lower income countries during the COVID‐19 pandemic.

**Key messages**

- Rapid household food insecurity (HFI) tracking has been identified as a priority in the context of the COVID‐19 pandemic and its aftermath.
- Cognitive testing and psychometric validation of the self‐administered HFI experience‐based ELCSA scale were conducted among pregnant women in Sri Lanka.
- The self‐administered ELCSA pregnancy was valid and feasible to efficiently track HFI during pregnancy in Sri Lanka.
- We recommend ELCSA pregnancy to track HFI among pregnant women in lower income countries.

## 1 INTRODUCTION

In high‐income countries (HICs) as well as low‐ and middle‐income countries (LMICs), household food insecurity (HFI) has been associated with poorer maternal and child health outcomes. HFI negatively impacts non‐communicable disease outcomes in pregnancy such as gestational diabetes mellitus, pregnancy‐induced hypertension and obesity (Hojaji et al., 2015; Laraia et al., 2010). A recent systematic review in South Asia indicated that HFI was a determinant of gender bias in intra‐household food allocation. Specifically, women's diets were compromised first when the household did not have adequate food access (Harris‐Fry et al., 2017). In LMIC's, HFI has been associated with child malnutrition and infectious diseases (Pérez‐Escamilla, 2017; Pérez‐Escamilla et al., 2009). HFI has indeed been associated with discrimination of women in the household, suggesting that women are the most vulnerable when households face food insecurity (Panter‐Brick & Eggerman, 1997). HFI has also been consistently associated with maternal anaemia, domestic violence and depression in women and poor early childhood development (Pérez‐Escamilla, Vilar‐Compte, et al., 2020).

With the global health focus on the aftermath of the pandemic, food insecurity has been predicted to become the ‘sting in the tail of COVID‐19’ (Pérez‐Escamilla, Cunningham, et al., 2020; The Lancet Global Helath, 2018); hence, careful and efficient monitoring of HFI has become a global public health priority. In the context of social distancing measures during a public health emergency, it is important to validate self‐applied HFI scales in lower income settings. Although telephone interviews could also be an option, this is not always possible in rural areas where telephone services are unreliable and not widely available. Furthermore, self‐applied questionnaires are likely to reduce the interviewer and information biases associated with direct interviews (Food and Agricultural Organization, 2016).

The pandemic has become a strong challenge for achieving the Sustainable Developmental Goals, especially in LMICs where maternal and under‐five malnutrition, morbidity and mortality are expected to rise as a result of increase in poverty and disruption of primary health and related services (Roberton et al., 2020). Hence, global health authorities have advised countries to be alert and prepared for a COVID‐19‐undernutrition syndemic strongly rooted in social determinants of health (SDH) inequities (Cornia et al., 2020). In 2005, the World Health Organization Commission for Social Determinants of Health was established to understand and act on inequities in health (Marmot, 2005). One of the three recommendations of the commission to close the gap in a generation was ‘to measure and understand the problem and assess the impact of action’ on inequities in SDH (Report of the Commission on Social Determinants of Health & The World Health Organization, 2008). Given that ‘closing the gap in a generation’ would be a challenge with the current COVID‐19 pandemic, counties across the globe need to track HFI frequently as it is a crucial SDH. This information is necessary for equitable resource allocations through effective food and nutrition security actions.

HFI has been measured indirectly through indicators such as anthropometry, dietary intake surveys and household food expenditure. Experience‐based HFI scales directly capture the experience of a household food situation, by selecting a respondent who is cognizant of the food situation in the family. The lack of access to a healthy and nutritious diet as a result of poverty, social deprivation or situations such as natural or man‐made disasters is well captured through experience‐based HFI scales (Ballard et al., 2013). The evolution and validation of experience‐based HFI assessment tools have been well documented across world regions. The US Household Food Security Survey Module (US‐HFSSM, 1995; Food and Agricultural Organization, 2009), Household Food Insecurity Access Scale (HFIAS, 2007; Coates et al., 2011) and FAO's *Escala Latinoamericana y Caribeña de Seguridad Alimentaria* (ELCSA) and derived Food Insecurity Experience Scale (FIES) have been validated across a number of different settings and are now being used extensively to track HFI globally (Pérez‐Escamilla, 2017; Pérez‐Escamilla, Vilar‐Compte, et al., 2020). Research in Mexico has shown that ELCSA can be applied by an interviewer over the phone to reliably and efficiently track HFI during the COVID‐19 pandemic (Gaitán‐Rossi et al., 2021). However, as far as we know, experience‐based HFI scales have not been validated in a self‐administered form in LMICs, which is a major gap in the context of pandemics or other public health emergencies, such as COVID‐19, requiring social distancing measures.

The objective of this study was to test the validity of a self‐applied culturally adapted eight‐item version of ELCSA in a large population‐based cohort study of pregnant women in Sri Lanka (Agampodi et al., 2020) in the context of the COVID‐19 pandemic.

## 2 METHODS

We conducted cognitive and psychometric validation of the self‐administered ELCSA for pregnant women in Sri Lanka (ELCSA‐P‐SL). The study population included pregnant women in their third trimester (32–36 weeks of gestation) of pregnancy enrolled in the Rajarata Pregnancy cohort (RaPCo) in Anuradhapura district, Sri Lanka. Ethical clearance was obtained from the Ethics Review Committee of Faculty of Medicine and Allied Sciences, Rajarata University of Sri Lanka. The study was conceived in December 2019 and was carried out in February 2020, just before Sri Lanka started to be affected by the COVID‐19 lockdown. Following the initial assessment, the pregnant women reporting severe HFI were recruited for a financial assistance donor programme voluntarily mediated by the institution.

### 2.1 The context

Sri Lanka was categorized as an upper‐middle‐income country in 2019 with a Gross National Income (GNI) per capita of USD 4060 (World Bank, 2020). The average life expectancy at birth for Sri Lankans is 75.5 years (United Nations Population Division., 2019). The literacy rate of males and females are 93.6% and 91.7%, respectively (Department of Census and Statistics Ministry of National Policies and Economic Affairs, 2017). More than 90% of the population has access to safe drinking water and sanitation (Department of Census and Statistics Ministry of National Policies and Economic Affairs, 2017). The prevalence of poverty (i.e., the percentage of the population below the national poverty line) is reported as 4.1% (Central Bank Sri Lanka, n.d.). The food security status of the country is reported in a global survey in 2016, indicating that 7.1% and 17.7% of the population is affected by severe and moderate to severe HFI respectively (Food and Agricultural Organization, 2016).

The present study was conducted in Anuradhapura, the largest district in Sri Lanka (7,179 m^2^). The total population in the district is 886,945 of which 94.1% is rural (Department of Census and Statistics, 2015). In this district, more than 17,000 pregnant mothers are registered annually for antenatal care (Ministry of Healthcare and Nutrition Sri Lanka, 2018). Demographic and Health Survey (DHS) data showed that antenatal care coverage through the public health system is 100%. Of the females in the district, 90% have at least entered secondary level education. The ongoing prospective cohort study in Sri Lanka (RaPCo study) provided a unique opportunity to validate a self‐reported measure of HFI due to its relatively high maternal literacy rates, universal maternal and child health service coverage and focus in equity for women.

### 2.2 Cognitive validation

The 8 adult items of the English version of ELCSA was culturally adapted to the Sri Lankan context and translated into Sinhala and Tamil following a modified version of Sumathipala and Murray's method (Sumathipala & Murray, 2000). A panel comprised of eight panelists, who are fluent in both languages, translated and then assessed the adapted questionnaire. Each panellist was provided with the original questionnaire. The concept of food security including its dimensions, and the objectives of the study were explained by an investigator. In the first round, panellists recorded their own translations. Each of the original English version and its corresponding eight translations were discussed one at a time until consensus was reached. The items were further refined in two subsequent rounds.

Cognitive validation was performed through expert opinion with five local experts with diverse experiences in the fields of nutrition, scale development methodology, maternal health, SDH and public health. Necessary changes were made to incorporate the comments of the experts with the consensus of the investigators. Target group interviews were held with 10 pregnant women using the methods suggested by Bowden, Fox‐Rushby, Nyandieka, and Wanjau (2002) for cognitive validation. A three‐step process was followed. First, the investigators observed whether the participant could easily understand and provide an answer for each item. Then, the cognitive process of the participant was examined to find out whether the participants thinking matched the intended meaning of each scale's item. Then, the participants were asked whether they had alternative wording suggestions for each item. We ensured that the original meaning was preserved and that the wording was clear, simple and self‐explanatory to facilitate its self‐administration.

### 2.3 Psychometric validation

The cognitively validated ELCSA‐P‐SL was self‐administered by a consecutive sample of 269 pregnant women attending the RaPCo special clinics in Anuradhapura district, Sri Lanka, in February 2020 (Agampodi et al., 2020). Data were collected from 21 out of 22 public health administrative areas (Medical Officer of Health areas) in the district. Pregnant women filled the questionnaire themselves. Participants psychological distress was simultaneously assessed using the validated self‐administered Sinhala and Tamil versions of the General Health Questionnaire 12 (GHQ 12; Abeysena et al., 2014) in order to assess the concurrent validity of the new HFI scale. We assessed the internal consistency, psychometric properties (including model fit) using a one parameter logistic model (Rasch model analysis) using STATA Version 14. Item severity scores and infit statistics were calculated using WINSTEP software Version 4.3.4.

The Rasch model has been used extensively to evaluate the internal validity of HFI scales, including ELCSA (Perez‐Escamilla et al., 2008; Gaitán‐Rossi et al., 2021). The Rasch model provides estimates of the severity of items based on the observed values and determines the degree that those items ‘fit’ the Rasch model expectations. Individuals or households experiencing higher levels of food insecurity are expected to affirm more items on an HFI scale than those experiencing lower or no levels of food insecurity. The more items that are affirmed by an individual or household, the more severe the level of food insecurity is in the household. Lower severity values indicate that the item is less severe and is affirmed by more individuals, whereas higher severity values indicate an item is more severe and is affirmed by fewer individuals. The Rasch model helps to evaluate whether an item's severity and the actual individual's food security status match in the way they are expected to. Items are considered to ‘fit’ the expectations of the Rasch model when the infit value falls within the range of 0.7–1.3.

## 3 RESULTS

### 3.1 Changes made in cognitive validation

Experts and pregnant women agreed that the questions were clear to them and that the intended meaning of each item was preserved. Based on the consensus from the experts, the introduction of the questionnaire was adjusted to include lay language, which could be easily understood by rural women. It was clearly mentioned that the lack of access to food was being asked in the context of lack of socio‐economic resources and that it did not refer to food restrictions due to dieting, loss of appetite or pregnancy‐related symptoms such as nausea, vomiting or heart burn. To maintain the respondents' focus on the time period, the phrase ‘during the past three months’ was added at the beginning of each question. To ensure that the exact intended reasons of food insecurity were taken into account, the phrases such as ‘due to the above mentioned reasons’ or ‘due to less availability of food in your house’ were used in each and every scale question. Using these phrases repeatedly was designed to enable participants to remain focused on accurately answering the ELCSA‐P‐SL self‐administered questions.

### 3.2 Psychometric assessment

#### 3.2.1 Characteristics of the study sample

Of the 328 pregnant women attended the RaPCo special clinics during this period, 269 responded to the questionnaire; hence, the response rate was 82%. The sample characteristics with regard to ethnicity and the level of education were representative of the study population (Table 1).

**TABLE 1:** Characteristics of the study participants (*N* = 269 pregnat women in Sri Lanka)

Characteristic		Frequency	Percentage (%)
Age	18 or less	6	2.3
	19–35	247	93.6
	Above 35	11	4.2
Ethnicity	Sinhala	243	92
	Tamil	1	0.4
	Moor	19	7.2
	Other	1	0.4
Level of education	Primary only	2	0.8
	Post‐primary	154	59
	Secondary	47	18
	Tertiary	58	22.2

#### 3.2.2 Frequency distribution of responses

Inability to have a diversity of food due to lack of money or other resources was the item more frequently affirmed. There were no pregnant women in this sample who affirmed Item 8; that is, not eating throughout the day (Table 2). The percentage of missing data ranged from 0.4% to 3.3% in each item. The missing data were replaced by the answer ‘no’, as it did not change the order of severity by doing so and it was predictable based on the pattern of responses in rest of items. As an example, a mother who responded ‘no’ to Item 7 (experience of hunger for one meal) would invariably not left with hunger during the whole day which indicates a ‘no’ answer for Item 8. Further assessment of model fit without replacing the missing data indicated no significant change in severity scores and infit values.

**TABLE 2:** Frequency distribution of responses to the ElCSA‐Prgenancy Sri Lank scale (ELCSA‐P_SL, *N* = 269)

Item	Answered “yes”	Percentage “yes”	Answered “no”	Percentage “no”	Missing data
Fear of run out of food	21	7.8%	245	91.1%	1.1%
Run out of food	16	5.9%	253	93.7%	0.4%
Run out of nutritious food	18	6.7%	242	90.0%	3.3%
Run out of food diversity	31	11.5%	234	87.0%	1.5%
Have skipped one meal	7	2.6%	260	96.7%	0.7%
Have reduced amount	9	3.5%	260	96.5%	1.9%
Experienced hunger for one meal	3	1.1%	263	97.8%	1.1%
Experienced hunger whole day	0	0%	267	100%	0.7%

#### 3.2.3 Psychometric properties

**FIGURE 1:**
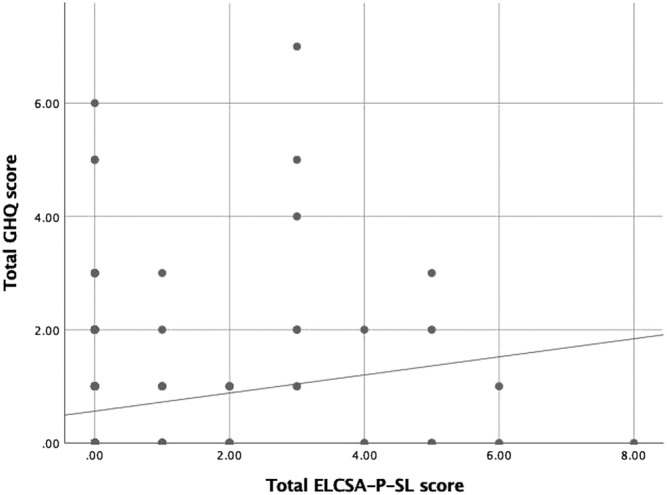
Scatter plot indicating a positive correlation between HFI and psychological distress (*N* = 269 pregnant women in Sri Lanka)

**FIGURE 2:**
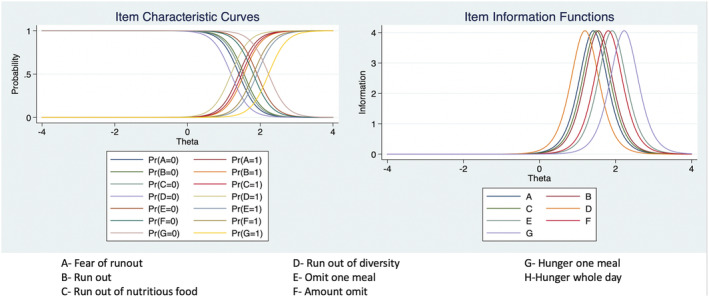
Item characteristic and information functions of ELCSA‐P‐SLa (*N* = 269 pregnant women in Sri Lanka)

**FIGURE 3:**
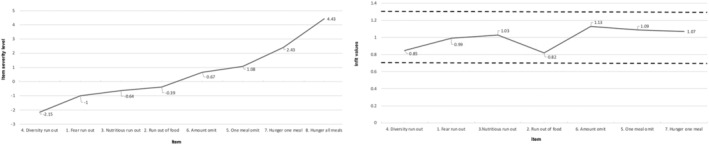
Item severity and infit statistics of ELCSA‐P‐SL (*N* = 269 pregnant women in Sri Lanka)

The tool was internally consistent (Cronbach's alpha = 0.79). The one parameter logistic model indicated that dietary diversity was the first factor to be compromised and that experience of hunger represented the most severe stage of HFI (Figure 1). The item infit statistics indicated good model fit (range: 0.85 to 1.07). Item 8 (did not eat for the whole day) was removed from the model fit analysis as none of the pregnant women affirmed this item. Overall, the item severity scores ranged from −2.15 for diversity food to 4.43 for hunger during the whole day. The item severity in Rasch model is based on the assumption that the log‐odds of a household affirming an item is proportional to the difference between the “true” severity of food security in the household and the “true” severity level represented by the item (Nord, 2014). The range of item severity was 6.28, and the ordering of severity (Figure 2) was as expected with the food diversity being the least severe and the item about not eating during the whole day the most severe. We observed a week positive (*r* = 0.15, *p* < 0.05) correlation between HFI and distress scores (Allen et al., 2018; Becerra et al., 2015) confirming the concurrent validity of the tool (Figure 3).

## 4 DISCUSSION

This study reports the validation of the eight‐item self‐administered ELCSA‐P‐SL (Data S1). The scale is pragmatic as it was easily self‐administered with minimal missing data. Furthermore, it is valid because it was judged by participants and experts to have strong face and content validity and adequate psychometric properties as demonstrated by Rasch analyses. Hence, the scale has the potential to be useful for rapid HFI tracking during pregnancy in Sri Lanka, which has become especially important under public health emergencies that require social distancing, such as the COVID‐19 pandemic. Although the ELCSA tool had been previously validated among pregnant adolescents in Brazil (Muñoz‐Astudillo et al., 2010), to the best of our knowledge, this is the first time that ELCSA was validated as a self‐administered questionnaire among pregnant adult women in a LMIC.

The severity scores of ELCSA‐P‐SL behaved as expected indicating that dietary diversity is compromised first and that food intake reduction or hunger is the most severe manifestation of HFI. Poor diet diversity has indeed been identified as being prevalent in Sri Lanka (Aguayo, 2017; Sirasa et al., 2020). In contrast to other contexts, being worried or having fear about running out of food is the item that has been more frequently affirmed (Pérez‐Escamilla, 2017; Pérez‐Escamila et al., 2017). Identification of possible underlying reasons for this observed inter‐country difference in items' ranking order requires further research.

This study is likely to have minimized social desirability bias as the HFI questionnaire was self‐administered (Food and Agricultural Organization, 2016). Likewise, pregnant women who may have felt shy or uncomfortable reporting deficiencies in household food availability in a face‐to‐face interview might have reported more accurate details in this self‐administered scale.

One important advantage of ELCSA is that it not only captures the access to food but also reflects the underlying pain and anxiety experienced by the participants due to food insecurity and hunger. Indeed, HFI derived from ELCSA‐P‐SL was significantly correlated with psychological distress, which is consistent with recent findings (Allen et al., 2018).

Ideally, the psychometric validation of ELCSA‐P‐SL should have been done separately for the Sinhala and Tamil language versions. However, because the number of Tamil pregnant women in the sample was very small (*n* = 20), we were not able to do so. Nonetheless, we confirmed that removing Tamil participants from the validation analysis led to very similar findings (available upon request from authors) and did not affect any of the conclusions.

The items included in the ELCSA‐P‐SL scale are not pregnancy specific; thus, in principle, this scale could also be used for rapid assessment of HFI in the lactation period or in the general population. Hence, further research is needed to assess the external validity of self‐administered ELCSA‐P‐SL to other stages of the woman's life course and/or other populations in the context of emergencies that require social distancing measures. In conclusion, ELCSA‐P‐SL can be self‐applied for assessing HFI during pregnancy in lower income countries. It is a timely contribution because it can be used to document and respond to increases in HFI during the COVID‐19 and other public health emergencies in the future.

## 5 CONCLUSION

The self‐administered ELCSA‐P‐SL is valid and feasible to rapidly track HFI among pregnant women in Sri Lanka.

## CONFLICTS OF INTEREST

The authors declare that they have no conflict of interest.

## CONTRIBUTIONS

RPE, TCA, SBA, GSA and IUJ designed the study. GSA, IUJ, NDW and AUH conducted the research. TCA, SBA and AHF conducted statistical analysis. TCA, NDW and AHF wrote the paper. RPE and SBA edited the manuscript. All authors had the primary responsibility for final content of the data. All authors have read and approved the final manuscript.

## Supporting information


**Data S1.** Supporting Information

## Data Availability

The data that support the findings of this study are available from the corresponding author upon reasonable request.
