# Supplementary material for: A self‐applied valid scale for rapid tracking of household food insecurity among pregnant women in Sri Lanka
Source: Matern Child Nutr. 2021 Mar 17;17(3):e13165. doi: 10.1111/mcn.13165 (PMC8189217; doi:10.1111/mcn.13165)
Supplement: Supplementary file 1 — Data S1. Supporting Information [file MCN-17-e13165-s001.docx]

**The Sri Lankan *Escala Latinoamericana y Caribena de Seguridad Al- imentaria -*ELCSA for pregnancy (ELCSA-P-SL)**

**Assessment of household food insecurity**

Our intention is to explore the food security in your house hold. To be simple we will assess whether all the members in your household have access to adequate amount of nutritious food. **It is believed that running out of money, inability to produce your own food, limited access to food in your village, poor social connections, lack of food subsidies, drought, lack of irrigation systems or political instability can give rise to food insecurity in your household.**

Please consider that “members in your household” in this questionnaire refers to only adults of more than 18 years of age and that children are not included. Please not that If yourself or a member of your household do not get adequate amount of food or nutritious food due to reasons other than above such as dieting, having the intention to reduce weight or poor appetite, it is not intended in this assessment. Similarly, if you are unable to take adequate amount of food or nutritious food due to symptoms of pregnancy such as nausea, vomiting and heart burn please note that it is not intended in this assessment. In fact, the reason for limitation of food or remaining in hunger should be due to poor access to food due to fore mentioned reasons such as poverty.

(Please note that you will not be deprived of any food subsidies or aids that you currently receive due to the answers you provide in this questionnaire. Neither you will be offered any new food subsidies and aids depending on the answers that you provide. As this effort is to generate information for the benefit of mothers and children in the future, we hope that you will provide us with the true information pertaining to your household.

**Please circle the correct answer for the following questions pertaining to the situation of access to food in your household within the last three months.**

| 1 | During the past three months, were there times when you were in fear and worry of the fact that your household would run out of food? | Yes | No | Don’t know |
| --- | --- | --- | --- | --- |
| 2 | During the past three months, did your household run out of food at any time? | Yes | No | Don’t know |
| 3 | During the past three months, due to above mentioned reasons, were you or other member in your household could not have a healthy and nutritious diet? | Yes | No | Don’t know |
| 4 | During the past three months, due to above mentioned reasons, were you or other member in your household had to eat the same food often restricting diversity? | Yes | No | Don’t know |
| 5 | During the past three months, did any day, you or any other adult in your home skipped breakfast, lunch or dinner because there wasn’t enough food in the household? | Yes | No | Don’t know |
| 6 | During the past three months did any adult in your home eat less food than what you think he/she needed because there wasn’t enough food in the household? | Yes | No | Don’t know |
| 7 | During the past three months was there any day when you or any other adult in your home felt hungry but did not eat because there wasn’t enough food? | Yes | No | Don’t know |
| 8 | During the past three months was there any day when you or any other adult in your home kept feeling hungry but didn’t eat for a whole day there wasn’t enough food? | Yes | No | Don’t know |
